# Supplementary figures and images for: Timing of integration into the chromosome is critical for the fitness of an integrative and conjugative element and its bacterial host
Source: PLoS Genet. 2023 Feb 13;19(2):e1010524. doi: 10.1371/journal.pgen.1010524 (PMC9956884; doi:10.1371/journal.pgen.1010524)

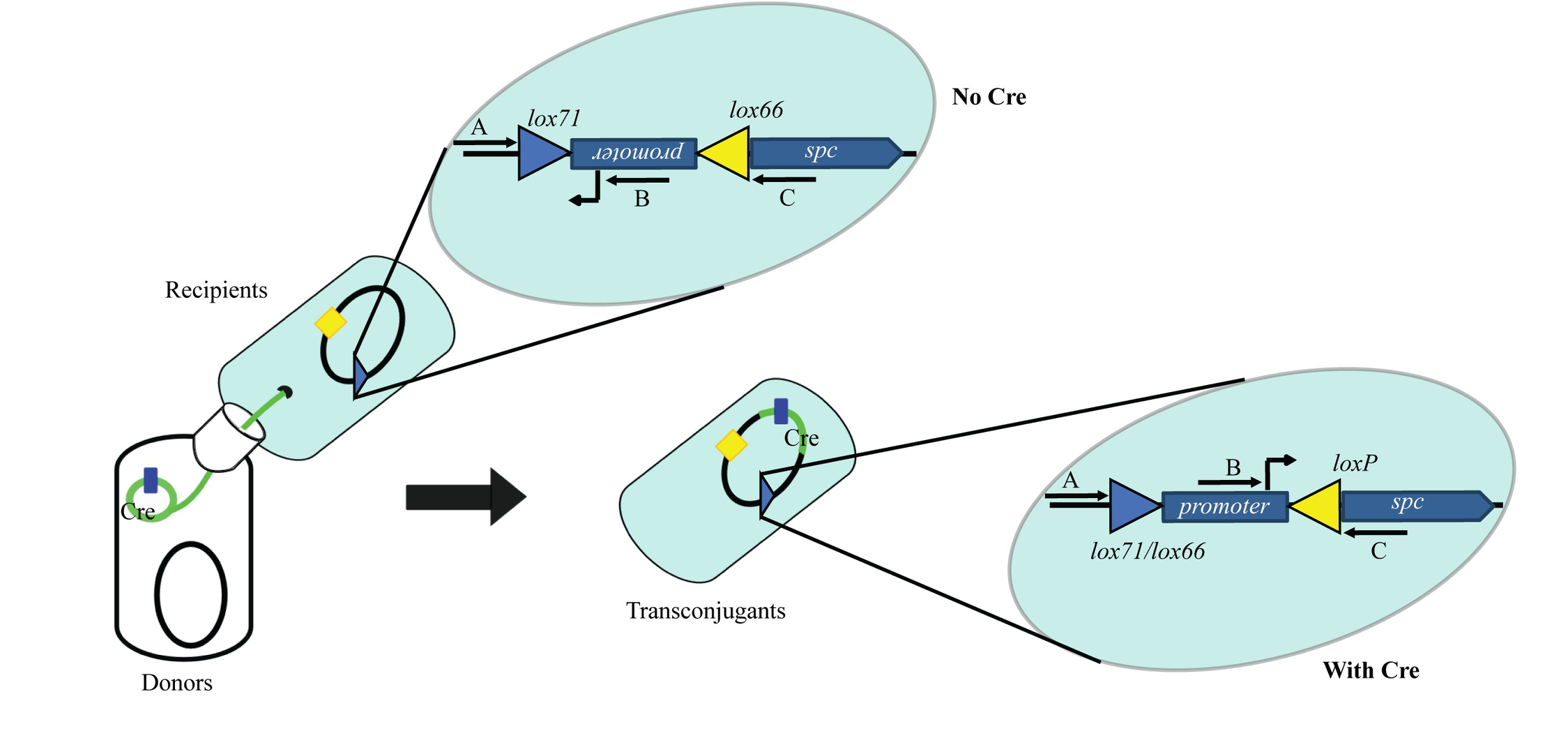

Supplement: S1 Fig — Donors containing ICEBs1 with Pxis-cre were mated with recipients harboring a reporter to indicate Cre-mediated recombination (inversion). Transfer of ICEBs1 with Pxis-cre and production of Cre results in inversion of the DNA fragment that can be detected by qPCR. The green circle in the cartoon at the bottom left represents ICEBs1 that expresses cre. The blue triangle in recipients and transconjugants indicates the reporter for and result of Cre-mediated inversion. Blue and yellow triangles indicate lox sites as labeled. The region between the blue and yellow triangles contains a promoter, either driving transcription of spc (With Cre; bottom right) or in the opposite orientation (No Cre; top). Blue block arrow represents spc open reading frame. Inversion in the presence of Cre produces lox71/lox66 and loxP sites which are incapable of subsequent recombination [64]. The horizontal black arrows with letters A, B, and C represent primers for PCR to detect the reporter and Cre-mediated inversion. PCR with primers B and C detect the product of Cre-mediated recombination. PCR with primers B and A detect the starting reporter with the promoter in the ’off’ orientation. (TIF) [file pgen.1010524.s001.tif]

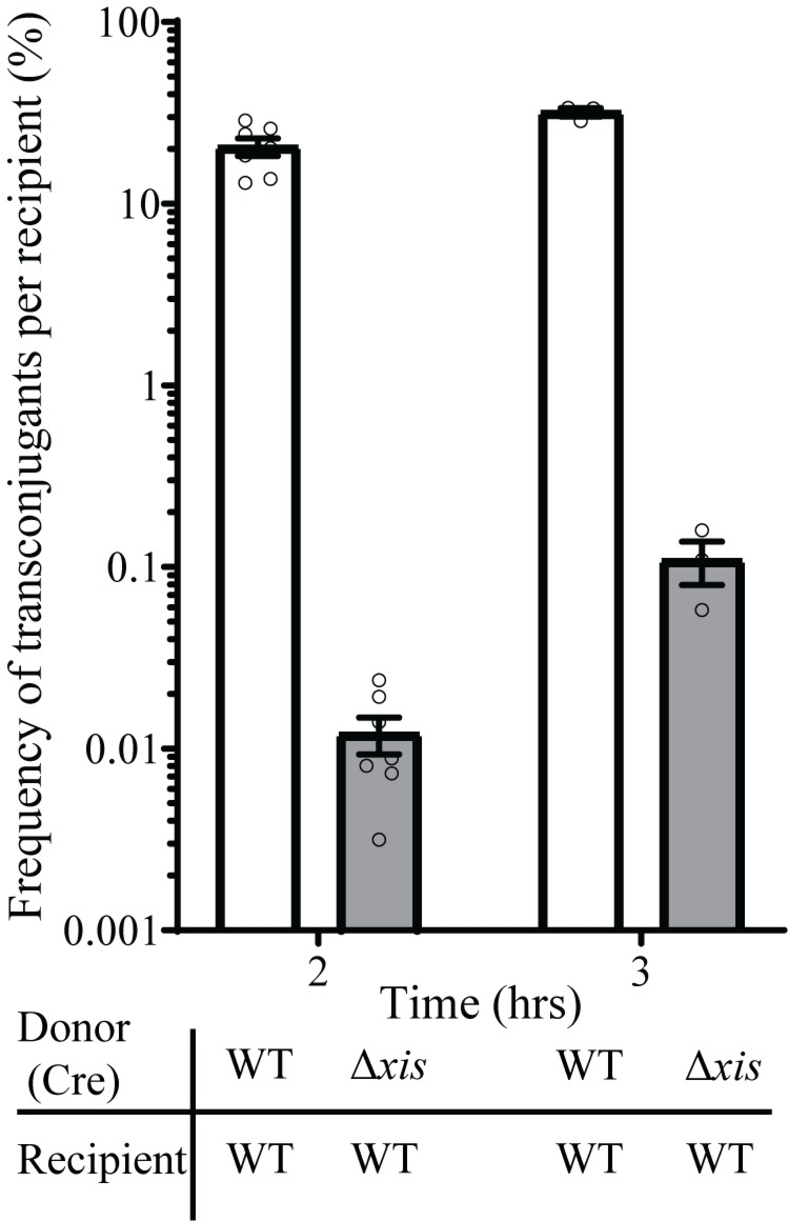

Supplement: S2 Fig — Acquisition of ICEBs1 by recipients was measured by selective plating and normalized to the total number of recipients. The identity of donor and recipient strain is indicated below the x-axis. Donors: WT (SAM830); Δxis (SAM892). Recipients: WT (SAM599). Circles indicate the results from independent experiments (at least three). Error bars indicate standard error of the mean. Results are from the averages of at least two technical replicates for CFUs. (TIF) [file pgen.1010524.s002.tif]

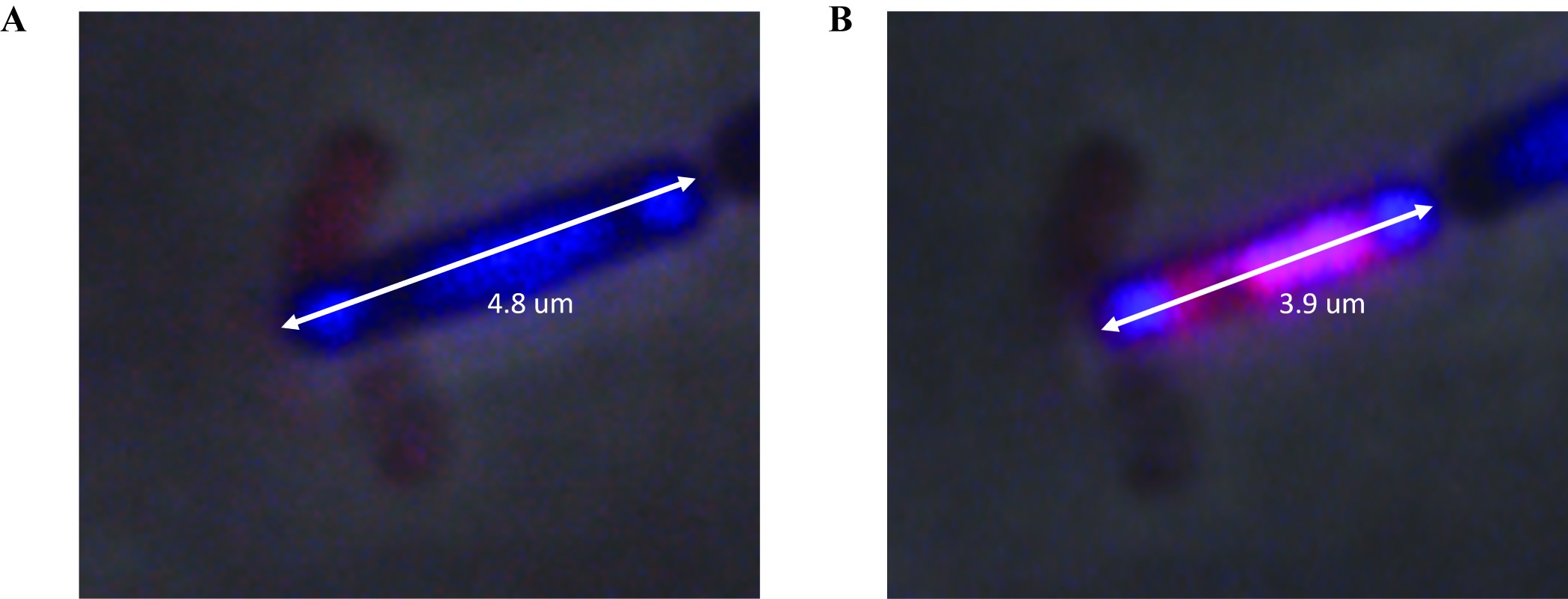

Supplement: S3 Fig — Cells were (ICEBs1 Δxis; SAM427) grown at 37°C in defined minimal medium with L-arabinose. Expression of rapI (Pxyl-rapI) was induced by the addition of D-xylose. After 60 mins, cells were washed and spotted onto agarose pads containing CH medium, DAPI, and propidium iodide. A) Cell 3 mins prior to PI uptake. The white arrows indicate the axis across which cell length was measured and the cell length is indicated under the arrow. DAPI is pseudo-colored blue. Propidium iodide is pseudo-colored red. B) Cell from A, at the time of uptake of propidium iodide and 3 mins prior to lysis. (TIF) [file pgen.1010524.s003.tif]

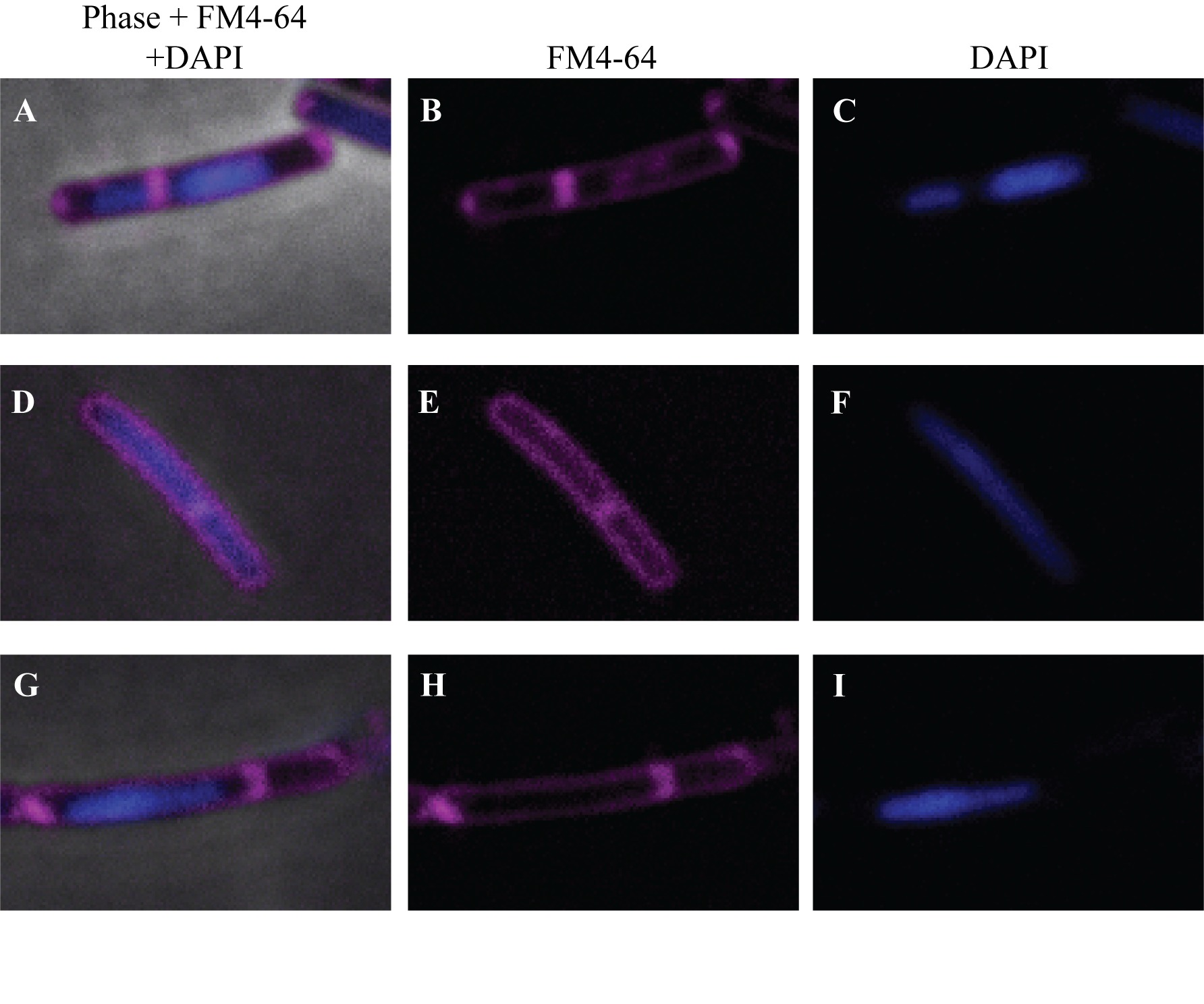

Supplement: S4 Fig — Cells (ICEBs1 Δxis; SAM427) were grown at 37°C in defined minimal medium with L-arabinose. Expression of rapI (Pxyl-rapI) was induced by the addition of D-xylose. After 60 mins, cells were washed and spotted onto agarose pads containing CH medium, DAPI, and FM4-64. A-C) Cell with asymmetric chromosome segregation and mislocalized septum formation. D-F) Cell with an elongated nucleoid and a guillotined chromosome. G-I) Cell with asymmetric chromosome segregation and anucleate division. A, D, and G) Composite image of phase, FM4-64 and DAPI channels. B, E, and H) FM4-64 channel. C, F, and I) DAPI channel. (TIF) [file pgen.1010524.s004.tif]

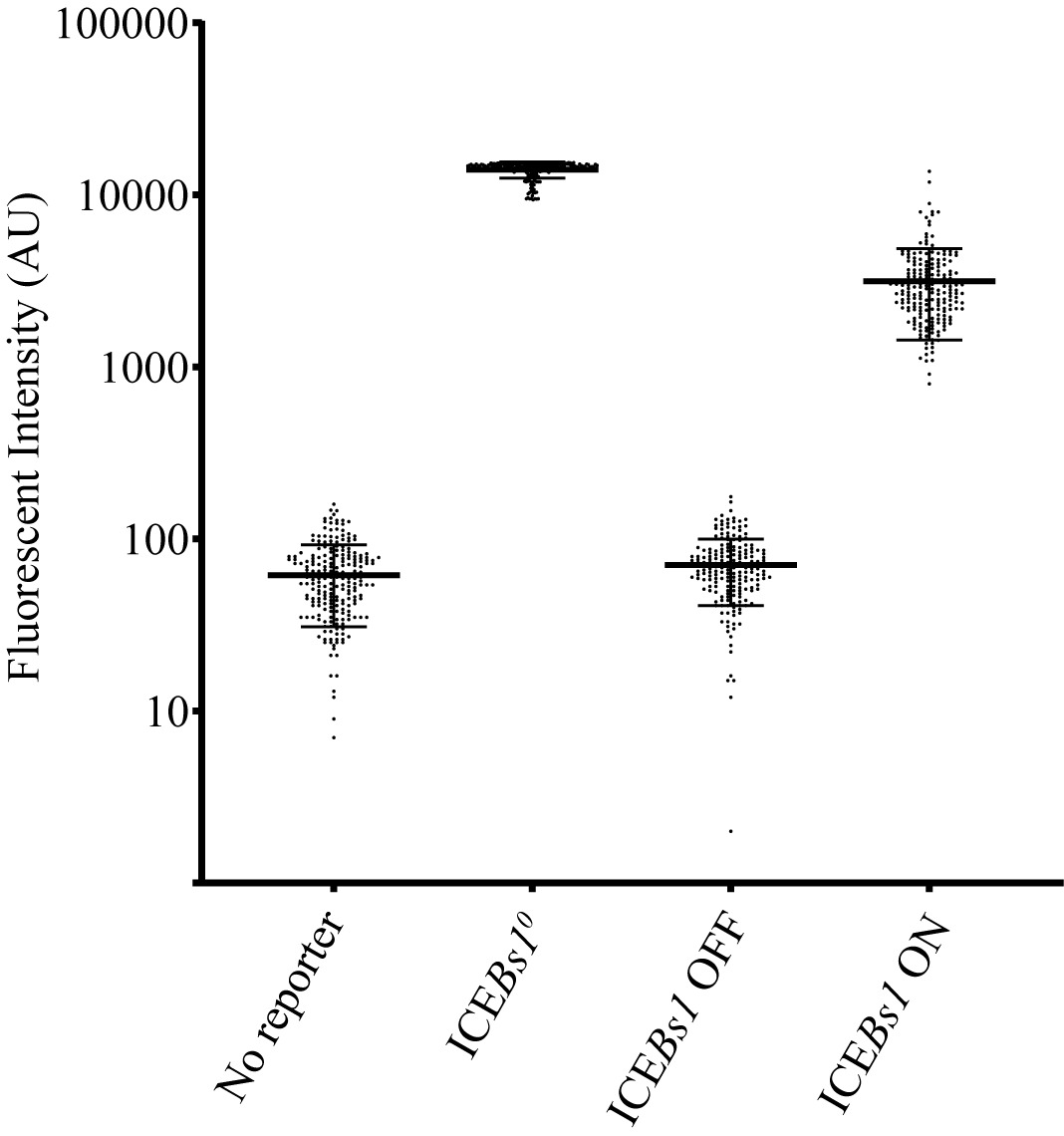

Supplement: S5 Fig — Cells with the reporter Pxis-gfp were grown at 37°C in a defined minimal medium with L-arabinose. Expression of rapI (Pxyl-rapI) was induced by the addition of D-xylose. After 2 hrs cells were spotted onto agarose pads and imaged for fluorescence. Each data point represents the fluorescent intensity from a single cell. The longest horizontal line represents the mean and bars above and below represent the standard deviation. No reporter is the parental strain with no genetic markers (AG174). ICEBs10 is a strain cured of ICEBs1 (SAM011) and expression from Pxis is in the absence of the repressor ImmR. ICEBs1 OFF is a strain containing ICEBs1 Δ(rapI-phrI)::kan (SAM032) without exogenous activation of the element. These cells produce active ImmR and transcription from Pxis is repressed and there is only basal expression of ICEBs1. ICEBs1 ON is the same strain (SAM032) except that ICE has been activated by virtue of expression of rapI and subsequent inactivation of the repressor ImmR. Pxis is expressed under these conditions. (TIF) [file pgen.1010524.s005.tif]

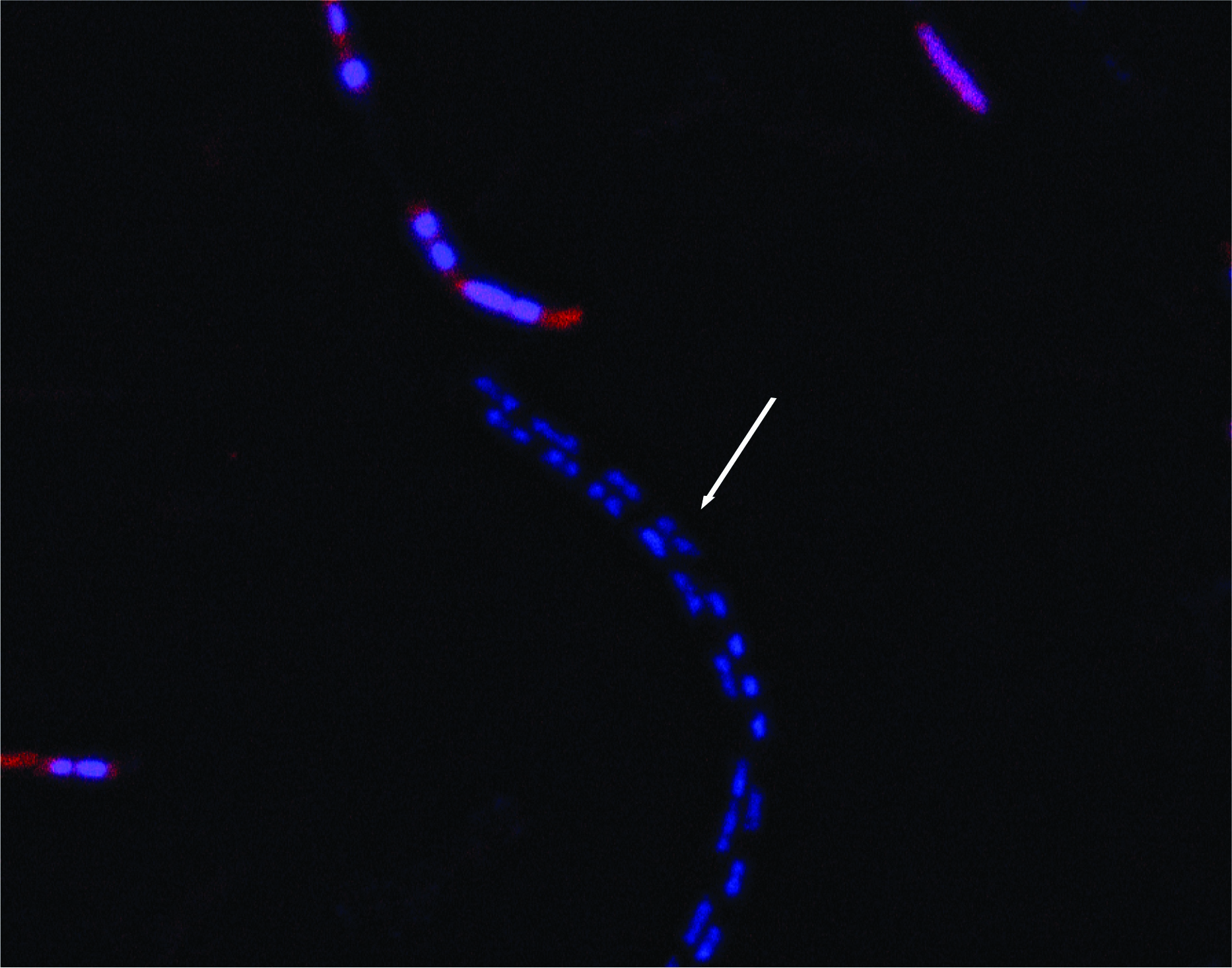

Supplement: S6 Fig — Cells containing ICEBs1 Δxis and the reporter Pxis-mApple (SAM456) were grown at 37°C in a defined minimal medium with L-arabinose. Expression of rapI (Pxyl-rapI) was induced by the addition of D-xylose. After 60 mins cells were washed and spotted onto agarose pads with CH medium and DAPI. The image shown is after 3 hrs on the agarose pad and is a composite of the red fluorescence (Pxis-mApple) and DAPI channels. Red fluorescence is pseudo-colored red and DAPI is pseudo-colored blue. The white arrow points to chain of normally growing cells that have ICEBs1 but the element has not been activated. Had they lost ICEBs1, or had ICEBs1 been activated, then Pxis-mApple would be expressed. (TIF) [file pgen.1010524.s006.tif]
